# Novel infusion strategy reduces severe adverse events caused by the anti-GD2 monoclonal antibody naxitamab

**DOI:** 10.3389/fonc.2023.1164949

**Published:** 2023-05-05

**Authors:** Amalia Varo, Alicia Castañeda, Saray Chamorro, Juan Pablo Muñoz, Maite Gorostegui, Mónica S. Celma, Sandra Lopez, Margarida Simao, Sara Perez-Jaume, Jaume Mora

**Affiliations:** ^1^ Pediatric Cancer Center Barcelona (PCCB), Hospital Sant Joan de Déu, Barcelona, Spain; ^2^ Department of Pharmacy, Pediatric Cancer Center Barcelona (PCCB), Hospital Sant Joan de Déu, Barcelona, Spain

**Keywords:** neuroblastoma, immunotherapy, adverse events, GD2 monoclonal antibody, naxitamab, pharmacokinetics, pharmacodynamics

## Abstract

**Introduction:**

Anti-disialoganglioside 2 (anti-GD2) monoclonal antibodies (mAbs) are associated with Grade ≥3 (≥G3) adverse events (AEs) such as severe pain, hypotension, and bronchospasm. We developed a novel method of administering the GD2-binding mAb naxitamab, termed “Step-Up” infusion (STU), to reduce the risk of AEs of severe pain, hypotension, and bronchospasm.

**Methods:**

Forty-two patients with GD2-positive tumors received naxitamab under “compassionate use” protocols and administered *via* either the standard infusion regimen (SIR) or the STU regimen. The SIR comprises a 60-min infusion of 3 mg/kg/day on Day 1 of cycle 1 and a 30- to 60-min infusion on Day 3 and Day 5, as tolerated. The STU regimen uses a 2-h infusion on Day 1, initiated at a rate of 0.06 mg/kg/h during 15 min (0.015 mg/kg) and which increases gradually to a cumulative dose of 3 mg/kg; on Days 3 and 5, the 3-mg/kg dose is initiated at 0.24 mg/kg/h (0.06 mg/kg) and delivered in 90 min according to the same gradual-increase strategy. AEs were graded according to Common Terminology Criteria for Adverse Events version 4.0.

**Results:**

The frequency of infusions with an associated G3 AE was reduced from 8.1% (23/284 infusions) with SIR to 2.5% (5/202 infusions) with STU. The odds of an infusion being associated with a G3 AE reduced by 70.3% with STU vs. SIR (odds ratio: 0.297; *p* = 0.037). Mean serum naxitamab levels pre- and post-STU (11.46 µg/ml pre-infusion; 100.95 µg/ml post-infusion) were within the range reported for SIR.

**Discussion:**

The comparable pharmacokinetics of naxitamab during SIR and STU may indicate that switching to STU reduces G3 AEs without impact on efficacy.

## 1 Introduction

Anti-disialoganglioside 2 (anti-GD2) monoclonal antibodies (mAbs) are an important treatment modality for patients with high-risk neuroblastoma (HR-NB), both in the frontline setting and for those with refractory or relapsed (R/R) disease (1–5). Naxitamab (hu3F8) is a humanized mAb approved for use in combination with granulocyte-macrophage colony-stimulating factor (GM-CSF) in pediatric (age >1 year) and adult patients with R/R HR-NB in the bone and/or bone marrow (BM) and who have demonstrated a partial response (PR), minor response (MR), or stable disease (SD) following prior therapy (6). Accelerated approval was granted in November 2020 by the United States Food and Drug Administration. Naxitamab is administered without the need for overnight hospital stay (for the infusion) as a 30- to 60-min infusion (standard infusion regimen; SIR) on Days 1, 3, and 5 of each cycle (7, 8). This contrasts with treatment protocols for other approved anti-GD2 mAbs for HR-NB (dinutuximab and dinutuximab beta), which require longer infusion times: dinutuximab is administered over 10–20 h for four consecutive days; dinutuximab beta is administered over 8 h for 5 days, or *via* continuous infusion with a portable pump for 10 days (9, 10). Naxitamab is similar to other approved anti-GD2 mAbs in terms of type of associated adverse events (AEs), which primarily occur during the treatment infusion and include pain, hypotension, hypertension, and bronchospasm (9–14). Most patients treated with naxitamab experience mild-to-moderate AEs, which are defined as Common Terminology Criteria for Adverse Events (CTCAE) Grade 1 or 2 (G1, G2); however, many patients also experience severe AEs (mostly CTCAE Grade 3; G3).

During naxitamab SIR (used during Trial 201; NCT03363373), G3 pain was experienced by 54% of patients, G3 hypotension by 59% of patients, and G3 bronchospasm by 18% of patients (see **Supplementary Table 1**) (8). G3 AEs such as hypotension and bronchospasm are medically significant events that may complicate treatment or result in hospitalization. While G3 pain may not pose the same clinical risks, it is nonetheless challenging for clinicians to manage and distressing for patients and caregivers.

Naxitamab infusion-related severe hypotension and bronchospasm are usually manageable with the use of normal saline bolus and a β2 receptor agonist, respectively; however, infusion-rate reductions or pauses may also be helpful (11). Pre-hydration with normal saline is recommended in the Trial 201 protocol to mitigate the risk of severe hypotension. In addition, premedication with a corticosteroid on Cycle 1, Day 1 is recommended to reduce the risk of severe bronchospasm and other potential infusion-related reactions. Based on the observation that the intensity of anti-GD2 mAbs-induced reactions diminishes over subsequent infusions (tachyphylaxis), we developed a novel infusion regimen for naxitamab, termed the “Step-up” infusion (STU) regimen, established on a pharmacodynamically adaptive sigmoid curve administration of the drug. This regimen aims to reduce the likelihood of a G3 AE (such as severe hypotension or bronchospasm) occurring during infusion, while maintaining efficacy. STU, which is designed to deliver a cumulative dose (3 mg/kg/day) *via* a stepwise approach and using a slower initial infusion rate, aims to modulate the pharmacodynamics of naxitamab. Herein, we report on safety outcomes from patients treated with SIR and STU regimens at HSJD.

## 2 Materials and methods

### 2.1 Study participants

Patients with GD2-positive tumors (HR-NB, retinoblastoma, and osteosarcoma) were included in the analysis. Patients were either in complete remission (CR), or had HR-NB with residual disease in the bone and/or BM compartment following multimodal or salvage therapy (i.e., refractory or relapsed disease) and were ineligible for Trial 201 (**Table 1**). Patients received naxitamab and GM-CSF under compassionate use between 7 December 2020 and 28 December 2021. Patients were deemed eligible for immunotherapy with naxitamab if major organ toxicity was of CTCAE ≤G2. Informed written consent for treatments and tests were obtained according to HSJD institutional review board rules. Treatment was planned to be administered without the need for overnight hospital stay during the infusion.

**Table 1:** Distribution of diagnoses per infusion administration cohort.

Diagnosis	SIR, n (%) [N = 18]	STU, n (%) [N = 11]	SIR and STU, n (%) [N = 13]
HR-NB (1st CR)	8 (44)	6 (32)	7 (58)
HR-NB (2nd CR)	5 (27)	2 (18)	2 (15)
HR-NB (3rd CR)	1 (6)	0 (0)	0 (0)
Primary refractory NB	3 (17)	0 (0)	2 (15)
Secondary refractory NB	0 (0)	1 (9)	2 (15)
Retinoblastoma (2nd CR)	1 (5)	0 (0)	0 (0)
Osteosarcoma (2nd CR)	0 (0)	1 (9)	0 (0)
CNS relapse of NB	0 (0)	1 (9)	0 (0)

CNS, central nervous system; CR, complete remission; HR-NB, high-risk neuroblastoma; n, number of patients per diagnosis; N, total number of patients; NB, neuroblastoma; SIR, standard infusion regimen; STU, “Step-Up” infusion regimen.

### 2.2 Study design and procedures

All patients were managed by the same team of nurses and physicians regardless of the infusion type. During the first half of the year 2021, infusions were administered following the SIR. While the STU was being developed, patients experiencing G3 AEs on the SIR were progressively moved to the STU and intrapatient comparisons were made for those having received both types of infusion. Over the course of the second half of the year, and given the improvements observed with the STU, patients’ infusions were progressively moved to the STU. As previously described (5), the infusion team consisted of three nurses: two to prepare the infusion suite before the patient arrived and to receive patients and families upon arrival, while the third nurse verified all the medications and doses, prepared the medications for each infusion, and ensured that the medications were available in the infusion suite. Throughout the infusions, two of the nurses were present at the patient’s bedside together with an infusion medical doctor. One nurse was responsible for the medication while the other nurse monitored vital signs and completed any registration paperwork. The third nurse remained outside the infusion suite and was responsible for post-naxitamab infusion patient monitoring/follow-up and for providing premedication to patients awaiting naxitamab infusion.

Per SIR protocols (as used in Trial 201), naxitamab was administered on Days 1, 3, and 5 of each treatment cycle at a dose of 3 mg/kg/day. The first infusion of naxitamab (Cycle 1, Day 1) was administered intravenously (i.v.) over 60 min; subsequent infusions were administered over 30–60 min, as tolerated.

Infusions following STU protocols were initiated at a rate of 0.06 mg/kg/h for 15 min (0.015 mg/kg) naxitamab on Day 1. The rate was then doubled every 15 min, delivering a total dose of 3 mg/kg over 120 min (**Table 2**). On Days 3 and 5 of naxitamab administration, STU was initiated at a higher rate (0.24 mg/kg/h [0.06 mg/kg]), before the rate was doubled every 15 min up to a maximum rate of 6 mg/kg/h, delivering a total dose of 3 mg/kg in 90 min (**Table 2**).

Table 2“Step-Up” infusion regimen by infusion volume (50 ml, 75 ml, 100 ml, 125 ml, 150 ml, and 175 ml).

**Table:** 

	Infusion time, min	Dose, mg/kg	Infusion rate, mg/kg/h	Infusion rate, ml/h	Volume, ml	Infusion rate, ml/h	Volume, ml
Day 1				Infusion volume 50 ml		Infusion volume 75 ml	
1	15	0.015	0.06	1	0.25	1.5	0.38
2	15	0.03	0.12	2	0.50	3.0	0.75
3	15	0.06	0.24	4	1.00	6.0	1.50
4	15	0.12	0.48	8	2.00	12.0	3.00
5	15	0.24	0.96	16	4.00	24.0	6.00
6	15	0.48	1.92	32	8.00	48.0	12.00
7	32	2.05	3.84	64	34.24	96.0	51.36
**Total**	122	3.0			50.0		75.0
Days 3 and 5				Infusion volume 50 ml		Infusion volume 75 ml	
1	15	0.06	0.24	4	1.00	6	1.50
2	15	0.12	0.48	8	2.00	12	3.00
3	15	0.24	0.96	16	4.00	24	6.00
4	15	0.48	1.92	32	8.00	48	12.00
5	15	0.96	3.84	64	16.00	96	24.00
6	11	1.14	6.00	100	19.00	150	28.50
**Total**	86	3.0			50.0		75.0

**Table:** 

	Infusion time, min	Dose, mg/kg	Infusion rate, mg/kg/h	Infusion rate, ml/h	Volume, ml	Infusion rate, ml/h	Volume, ml
Day 1				Infusion volume 100 ml		Infusion volume 125 ml	
1	15	0.015	0.06	2	0.50	2.5	0.63
2	15	0.03	0.12	4	1.00	5	1.25
3	15	0.06	0.24	8	2.00	10	2.50
4	15	0.12	0.48	16	4.00	20	5.00
5	15	0.24	0.96	32	8.00	40	10.00
6	15	0.48	1.92	64	16.00	80	20.00
7	32	2.05	3.84	128	68.48	160	85.60
**Total**	122	3.0			100.0		125.0
Days 3 and 5				Infusion volume 100 ml		Infusion volume 125 ml	
1	15	0.06	0.24	8	2.00	10	2.5
2	15	0.12	0.48	16	4.00	20	5.00
3	15	0.24	0.96	32	8.00	40	10.00
4	15	0.48	1.92	64	16.00	80	20.00
5	15	0.96	3.84	128	32.00	160	40.00
6	11	1.14	6.00	200	38.00	250	47.50
**Total**	86	3.0			100.0		125.0

**Table:** 

	Infusion time, min	Dose, mg/kg	Infusion rate, mg/kg/h	Infusion rate, ml/h	Volume, ml	Infusion rate, ml/h	Volume, ml
Day 1				Infusion volume 150 ml		Infusion volume 175 ml	
1	15	0.015	0.06	3	0.75	3.5	0.88
2	15	0.03	0.12	6	1.50	7	1.75
3	15	0.06	0.24	12	3.00	14	3.50
4	15	0.12	0.48	24	6.00	28	7.00
5	15	0.24	0.96	48	12.00	56	14.00
6	15	0.48	1.92	96	24.00	112	28.00
7	32	2.05	3.84	192	102.72	224	119.84
**Total**	122	3.0			150.0		175.0
Days 3 and 5				Infusion volume 150 ml		Infusion volume 175 ml	
1	15	0.06	0.24	12	3.00	14	3.50
2	15	0.12	0.48	24	6.00	28	7.00
3	15	0.24	0.96	48	12.00	56	14.00
4	15	0.48	1.92	96	24.00	112	28.00
5	15	0.96	3.84	192	48.00	224	56.00
6	11	1.14	6.00	300	57.00	350	66.50
**Total**	86	3.0			150.0		175.0

Naxitamab cycles started with subcutaneous (s.c.) GM-CSF for 5 days at 250 μg/m^2^/day before the first dose of naxitamab (Days −4 to 0); GM-CSF at 500 μg/m^2^/day s.c. was then administered on Days 1–5, with GM-CSF administered at least an hour before naxitamab on infusion days. GM-CSF was not given if the absolute neutrophil count was >20×10^9^/L and/or white blood cell count was >50×10^9^/L. Treatment cycles of 9 mg/kg total naxitamab dose were repeated every 4 weeks for up to 7 cycles.

Pharmacokinetics were studied by quantifying serum naxitamab concentrations by enzyme-linked immunosorbent assay (ELISA) as previously reported for the SIR (15). Naxitamab serum levels were measured immediately before and 5 min after the end of STU over multiple cycles. Comparison of reported SIR and newly acquired STU naxitamab serum levels using the same methodology was performed. Infusion-related AEs were graded according to CTCAE version 4 (16) and managed as reported (5, 11).

### 2.3 Premedication and supportive treatments

All patients, regardless of the infusion protocol, received the same premedication with paracetamol (15 mg/kg oral, 1,000 mg max), cetirizine (<20 kg body weight: 2.5 mg oral; ≥20 kg body weight: 5 mg oral; age >12 years and >30 kg body weight: 10 mg oral), ranitidine (i.v. 1 mg/kg, 50 mg max), which was later changed to famotidine (0.5 mg/kg oral, 40 mg max) due to availability issues, and ondansetron (i.v. 0.15 mg/kg, 8 mg max) 30 min prior to the start the infusion. All patients on Cycle 1, Day 1 received methylprednisolone (i.v. 2 mg/kg; 80 mg max) per the standard Trial 201 protocol. For each infusion, patients followed pain management according to either regimen 1 (opioids only) or regimen 2 (ketamine-based) as described below:

#### 2.3.1 Regimen 1

Opioids only. Both SIR and STU: morphine chloride (i.v. 50 µg/kg, 4 mg max) 5 min before infusion and during infusion, if needed, for breakthrough pain.

#### 2.3.2 Regimen 2

Ketamine only. Approach differed slightly for SIR vs. STU. Initial combination for both was ketamine (i.v. 0.5–1 mg/kg) in combination with midazolam (i.v. 0.05 mg/kg, 2 mg max), atropine (i.v. 0.005 mg/kg, 0.6 mg max), and lidocaine (i.v. 2 mg/kg)—for SIR, this was given as premedication 3–5 min before the infusion, whereas for STU, it was given as supportive therapy when the first signs of pain appeared. For SIR, a second bolus of ketamine (dose as before) and lidocaine (1 mg/kg) was administered 15 min into the infusion. In both regimens, additional doses of ketamine (same dose, up to a total of 4 mg/kg) were administered as needed for breakthrough pain; in the STU group, this was supplemented by lidocaine (1 mg/kg, >15 min after the last dose) or midazolam (i.v. 0.05 mg/kg; total max of 4 mg), as required.

In this study, most patients began naxitamab treatment with STU, or switched to STU, after the ketamine-based regimen was established as the preferred pain management strategy for naxitamab infusions at HSJD. Therefore, most patients following STU protocols were given ketamine during infusions. All patients, regardless of pain-management regimen or infusion protocol, received dexketoprofen (1 mg/kg oral, 50 mg max) or metamizole (i.v. 20–30 mg/kg, 2,000 mg max) at the end of naxitamab infusion, if needed.

### 2.4 Statistical analysis

A logistic mixed model (estimated using maximum likelihood) was fit using the R (17) and lme4 (18) software packages to analyze the relationship between the type of infusion (SIR vs. STU) and the occurrence of G3 AEs. The model included individual and cycle number as random effects, and type of infusion (SIR vs. STU) as a fixed effect; odds ratios were derived from this model. The Wald approximation was used to compute 95% confidence intervals (CIs) and *p*-values (*p*-values <0.05 were considered statistically significant).

## 3 Results

### 3.1 Patients and infusions

Forty-two patients with GD2-positive tumors (HR-NB: *n* = 40; retinoblastoma: *n* = 1; osteosarcoma: *n* = 1) were treated with naxitamab (**Table 1**); 24 patients completed planned 5 cycles, 2 patients completed 7 cycles, and 16 patients received less than 5 cycles. Of these 42 patients, 18 followed SIR infusion protocols exclusively, 11 followed STU protocols exclusively, and 13 followed a mixture of both SIR and STU protocols. For patients who followed both SIR and STU protocols, most began naxitamab treatment with SIR and switched to STU for subsequent cycles as a strategy for mitigating severe AEs. However, seven patients were required to switch from STU to SIR for logistical reasons (not enough space for long infusions in the day hospital area).

A total of 486 naxitamab infusions were completed, 284 (58%) following SIR protocols and 202 (42%) following STU protocols. SIR was used for 73 infusions in Cycle 1 (25%), 71 in Cycle 2 (24%), 50 in Cycle 3 (18%), 45 in Cycle 4 (15%), 39 in Cycle 5 (13%), 3 in Cycle 6 (1%), and 3 in Cycle 7 (1%). STU was used for 47 (23%) infusions in Cycle 1, 40 (20%) in Cycle 2, 43 (21%) in Cycle 3, 33 (16%) in Cycle 4, 33 (16%) in Cycle 5, 3 (1.5%) in Cycle 6, and 3 (1.5%) in Cycle 7. Opioids were administered during 196 (69%) infusions that followed SIR protocols and 21 (10%) that followed STU protocols. Ketamine was administered during 88 (31%) infusions that followed SIR protocols and 181 (90%) that followed STU protocols.

### 3.2 Adverse events and infusions

All patients experienced at least one G1 or G2 AE during naxitamab infusion; 19 (45%) patients experienced at least one G3 AE. Of those patients who experienced at least one G3 AE, eight (42%) were following SIR protocols exclusively, two (11%) were following STU protocols exclusively, and nine (47%) were following both SIR and STU protocols. Of the nine patients following both protocols and who experienced at least one G3 AE, six (67%) experienced the G3 AE(s) during SIR, one (11%) experienced the G3 AE(s) during STU, and two (22%) experienced the G3 AE(s) during both SIR and STU.

A total of 23 episodes of G3 AEs occurred during SIR infusions, whereas a total of 5 episodes of G3 AEs occurred during STU infusions; G3 AEs were hypotension, hypertension, laryngospasm, bronchospasm, apnea, pain, and anaphylaxis (**Table 3**). Some patients experienced several G3 episodes during each cycle (three infusions/cycle) given either as SIR or STU. Seven patients experienced G3 hypotension episodes during SIR (37%) vs. two during STU (11%), and six patients experienced G3 hypertension episodes during SIR (32%) vs. two during STU (11%); no patients experienced both hypotension and hypertension at the same episode. Severe upper-airway compromise in the form of laryngospasm or bronchospasm episodes occurred in four patients each (21%; during SIR only), whereas apnea occurred in two patients (11%; one during SIR and one during STU), pain occurred in one patient (5%; during STU), and anaphylaxis occurred in one patient (5%; during SIR). None of the G3 AEs were life threatening or required hospitalization, irrespective of infusion regimen.

**Table 3:** Number of G3 AEs occurring during SIR and STU infusions by AE type and by cycle number.

	G3 AE episodes N = 28^a^	
	SIR n = 23	STU n = 5
By AE type, n (%)		
*Hypotension*	7 (37)	2 (11)
* Hypertension*	6 (32)	2 (11)
* Laryngospasm*	4 (21)	0
* Bronchospasm*	4 (21)	0
* Apnea*	1 (5)	1 (5)^b^
* Pain*	0	1 (5)^b^
* Anaphylaxis*	1 (5)	0
By cycle number, n (%)		
* 1*	14 (60)	2 (33)
* 2*	6 (28)	2 (33)
* 3*	2 (8)	1 (17)
* 4*	1 (4)	0

a 19 patients experienced a total of 28 G3 AE episodes.

b Apnea and pain occurred in the same G3 AE episode.

AE, adverse event; n, number of episodes on or more G3 AEs occurred; N, total number of episodes; SIR, standard infusion regimen; STU, “Step-Up” infusion regimen.

Thirty-one G3 AEs were reported across both infusion protocols from the total of 486 infusions; these AEs occurred during 28 infusions, giving an overall G3 AE frequency of ~6%. When naxitamab infusion was administered per SIR protocols, 25 G3 AEs occurred during 23 of 284 infusions, giving a G3 AE frequency of 8.1% for SIR. A lower frequency was observed for STU protocols, with a G3 AE reported in 5 of 202 infusions (giving a frequency of 2.5%; 1 infusion had two G3 AEs recorded at the same time, pain and apnea). The association of the type of infusion with the occurrence of a G3 adverse event was statistically significant (*p* = 0.037); the odds ratio for STU vs. SIR infusion was 0.297 (95% CI 0.095–0.929), representing a 70.3% reduction in the likelihood of a G3 AE with STU vs. SIR.

When considering AEs by infusion cycle, the majority of G3 AEs occurred during Cycle 1, with a decrease over subsequent cycles. For SIR protocols: 14 G3 AEs (60% of the total) occurred during Cycle 1, 6 (28%) occurred during Cycle 2, 2 (8%) occurred during Cycle 3, and 1 (4%) occurred during Cycle 4; there were no G3 AEs in subsequent cycles. However, for STU, the occurrence of G3 AEs during infusion did not appear to decrease over subsequent cycles: two (33%) G3 AEs occurred during Cycle 1, two (33%) occurred during Cycle 2, and one (17%) occurred during Cycle 3.

### 3.3 Pharmacokinetics and logistics related to the type of Infusion

**Figure 1:**
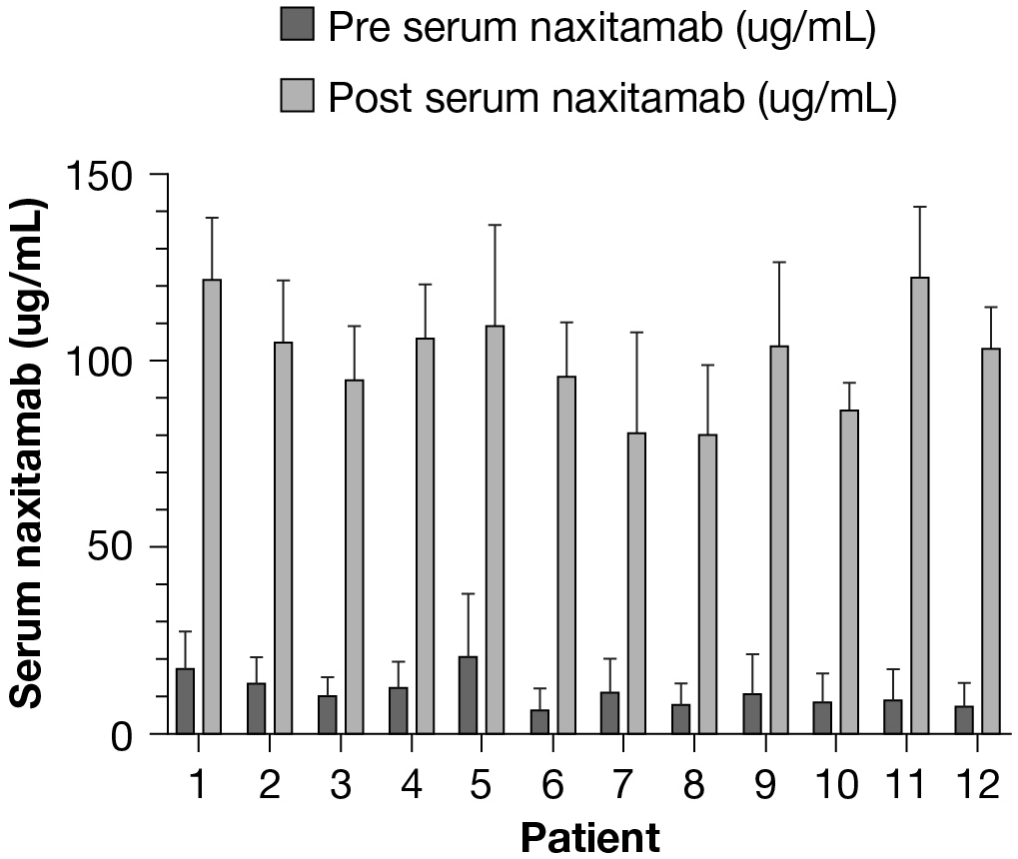
Pharmacokinetics of naxitamab on the “Step-Up” infusion protocol.

Naxitamab pharmacokinetics have been well described following SIR protocols (15). In order to investigate whether the STU regimen may interfere with drug distribution, we systematically collected serum to determine naxitamab levels pre- and post-infusion for all STU infusions. Examples from samples obtained from 12 patients over multiple cycles of STU pre- and post-infusion serum levels are shown in **Figure 1**. Overall, the average pre- and post-infusion serum naxitamab levels were 11.46 µg/ml and 100.95 µg/ml, respectively. No correlation with patient body weight was found. Post-infusion levels are within the same range (88–114 μg/ml) as those reported in the phase 1 Trial 12-230 study (NCT01757626; SIR only; 87.99 µg/ml at a total dose of 3.0 mg/kg) (15).

A reduction in the occurrence of G3 AEs when naxitamab was administered per STU protocols permitted a decrease in the intensity of patient monitoring required. As previously reported, with the SIR protocol, two nurses were required at the patient’s bedside together with the infusion physician (5). With the decrease in severity of the infusion-related reactions seen with STU protocols, only one bedside nurse was required, and a physician was no longer required to stay at the bedside. Pain management was still required during STU.

## 4 Discussion

The data presented in this analysis is based on patients treated with naxitamab under compassionate use at HSJD managed and assessed by the same team accustomed to evaluating naxitamab-related AEs. In the current study, STU (vs. SIR) protocols reduced the odds of G3 AEs occurring during infusion by 70.3% in patients with anti-GD2 positive tumors and who were in CR, and in patients with HR-NB with residual disease limited to the bone and/or BM following multimodal or salvage therapy (i.e., refractory or relapsed disease).

A similar approach to the STU regimen has been developed for bispecific antibodies like blinatumomab to prevent severe toxicities like the Cytokine Release Syndrome. The severe toxicity induced by blinatumomab has been mitigated by the administration of a stepwise dosing approach (19). By exposing the patient to a lower dose of naxitamab at the start of the infusion, STU has the potential to modify the pharmacodynamics of naxitamab, which may reduce associated toxicity. However, pain remains to be a notable AE and requires pre-medication and AE management during naxitamab infusion. Previous phase 1 studies (15, 20) have demonstrated a strong correlation between the peak serum concentration and area under the serum concentration time curve, which are measures of drug exposure over time and a critical determinant of mAb antitumor effect. Peak serum concentration of naxitamab is reached for an average of 5 min post-infusion (15). As naxitamab serum levels post-infusion for STU were similar to those previously reported during phase 1 studies using SIR protocols (15), drug exposure and antitumor effects should not be negatively affected by STU administration. This suggests that STU administration can reduce toxicity during naxitamab infusions without compromising efficacy.

As opioids can exacerbate hypotension, the type and timing of medication administered to manage pain (opioids only or a ketamine-based regimen) should be considered. Opioids are recommended for managing pain associated with naxitamab infusion (6) and are given to most patients treated using SIR protocols. The potential hypotensive effects of opioids may have contributed to the increased number of hypotension events seen in patients following SIR protocols vs. STU (seven for SIR vs. two for STU). Therefore, patients may have a lower risk of G3 hypotension when following STU protocols, partially due to following a ketamine-based regimen for pain management. However, it is also possible that the onset of hypotension may be slower with STU vs. SIR, allowing staff to prevent G1 or G2 hypotension from progressing to G3.

A physician and two nurses are normally required at the bedside for SIR, whereas only one nurse is required for STU. STU provides a clinically meaningful impact by reducing staffing needs vs. SIR. Furthermore, like SIR, STU has a short infusion time (1.5–2 h), allowing for treatment without the need for overnight hospital stay, providing convenience for patients and families.

In conclusion, STU naxitamab administration represents a novel way to deliver naxitamab treatment that may have the potential to reduce the likelihood of G3 AEs occurring during infusion. As the current analysis is retrospective, a prospective study is needed to establish whether STU protocols can deliver an improved AE profile for naxitamab vs. SIR. Establishing STU as the preferred method for naxitamab administration could optimize patient care and reduce both the care load for clinicians and staffing requirements for institutions.

## Data availability statement

The original contributions presented in the study are included in the article/**Supplementary Material**. Further inquiries can be directed to the corresponding author.

## Ethics statement

The studies involving human participants were reviewed and approved by Hospital Sant Joan de Deu. Written informed consent to participate in this study was provided by the participants’ legal guardian/next of kin.

## Author contributions

Conceptualization: JM. Data curation: AV and SP-J. Formal analysis: SP-J and JM. Funding acquisition: JM. Methodology: SC, JPM, AC, MG, MC, SL, and MS. Project administration: JM. Resources: JM. Software: SP-J. Supervision: JM. Validation: all authors. Writing—original draft: JM. Writing—review and editing: all authors. All authors contributed to the article and approved the submitted version.
