# Supplementary material for: Novel infusion strategy reduces severe adverse events caused by the anti-GD2 monoclonal antibody naxitamab
Source: Front Oncol. 2023 May 5;13:1164949. doi: 10.3389/fonc.2023.1164949 (PMC10196122; doi:10.3389/fonc.2023.1164949)
Supplement: Supplementary file 1 [file Table_1.docx]

**Supplementary table Summary of relevant naxitamab related treatment-emergent adverse events with a CTCAE Grade ≥3 from the first prespecified analyses of the pivotal 201 trial [8]**

| **Adverse event, n (%)** | **N=74** |
| --- | --- |
| Pain | 40 (54)^a^ |
| Hypotension | 44 (59)^b^ |
| Bronchospasm | 13 (18)^a^ |

^a^ No CTCAE Grade 4 was reported;
^b^ 3 patients (4.1%) reported 3 CTCAE Grade 4 events of hypotension.

CTCAE, Common Terminology Criteria for Adverse Events.
